# Characterization of immunologically detectable T-cell sensitization, Immunohistochemical detection of pro-inflammatory cytokines, and clinical parameters of patients after allogeneic intraoral bone grafting procedures: a prospective randomized controlled clinical trial in humans

**DOI:** 10.1186/s12903-022-02584-6

**Published:** 2022-12-10

**Authors:** Önder Solakoglu, Werner Götz, Volker von Baehr, Guido Heydecke, Klaus Pantel, Heidi Schwarzenbach

**Affiliations:** 1 The Dental Department of the University Medical Center Hamburg-Eppendorf, Martinistr. 52, 20246 Hamburg, Germany; 2 Department of Orthodontics, Laboratory for Oral Biologic Basic Science, University of Bonn, Bonn, Germany; 3 IMD Laboratory for Molecular Diagnostics, Berlin, Germany; 4 The Dental Department of Prosthodontics of the University Medical Center Hamburg-Eppendorf, Hamburg, Germany; 5 Department of Tumor Biology, University Medical Center Hamburg-Eppendorf, Hamburg, Germany

**Keywords:** Allogeneic bone graft, Lymphocyte transformation test, Human histology, Immunohistochemistry, Alloimmunization

## Abstract

**Background:**

The null hypotheses were tested that intraoral bone augmentation using two different allogeneic materials has no impact on the patient’s blood levels of material-specific lymphocytes and on the immunohistochemical detection of pro-inflammatory cytokines IL-1α, IL1ß and TNF-α and T-cell markers CD4, CD8 in biopsies of the test groups.

**Methods:**

In this prospective RCT, 60 systemically healthy participants were randomly assigned to two allogeneic test groups (1: Maxgraft®, freeze-dried, multiple donors, and 2: Puros®, solvent-dehydrated, single donor) and an autologous control group (10 patients). Plasma samples were collected pre-(T1) and postoperatively (2 weeks (T2) and 4 months (T3)). The Lymphocyte Transformation Test (LTT) was used for analyzing levels of transformed lymphocytes for type IV immune reactions by 3H-thymidine activity. Bone biopsies were harvested at T3 and immunohistochemically analyzed for IL-1α, IL1ß, TNF-α, CD4, CD8 and correlated with the immunological and clinical findings.

**Results:**

A statistically significant difference between the tested materials was observed for LTT measurements at T3 (*p* = 0.033). Furthermore, three groups were identified: Group A (LTT negative T1-T3, *n* = 48), group B (LTT positive T1-T3, *n* = 7), group C (developing positive LTT at T2, *n* = 5). A highly significant elevation of IL-1α, IL1ß, TNF-α in patients of group C (*p* = 0.0001) and a significant elevation of CD4+ cells in patients of group B (*p* = 0.005) was shown.

**Conclusion:**

Our data show that following allogeneic bone grafting, local and systemic immunological reactions can be detected in some patients. These findings were statistically significant for the timepoint T3 between the tested materials as well as for the groups B and C correlated with group A for both tested materials. Therefore, the null hypotheses were rejected. A preoperative compatibility test for allogeneic materials in order to improve patient safety and the predictability of these materials would be desirable.

**Trial registration:**

Ethical commission of the Ärztekammer Hamburg, Germany (PV5211) as well as by the German Registry of Clinical Studies (DRKS00013010) on 30/07/2018 (http://apps.who.int/trialsearch/).

**Supplementary Information:**

The online version contains supplementary material available at 10.1186/s12903-022-02584-6.

## Introduction

The use of dental implants for the replacement of missing natural teeth has become a safe and predictable treatment modality within the last decades [1, 2]. However, most commonly severe damage to the alveolar bone occurs following loss of natural teeth. This might be due to traumatic events as well as to inflammatory processes like periodontal diseases or resorptive processes resulting in vertical and / or horizontal bone defects [3–6]. Therefore, it is commonly required to perform bone augmentation procedures and to rebuilt the previously lost bone volume in order to allow for implant placement [7]. Numerous bone grafting materials for intraoral bone augmentation procedures are described in the literature and are widely used in oral surgery procedures [8]. As the gold standard for intraoral grafting procedures autologous bone is still considered the material of choice, however, its intraoral availability is limited and it is always associated with an intraoral or extraoral harvesting site, which could be associated with a significant postoperative morbidity [9–11]. Therefore, the use of bone grafting materials became commonly accepted in order to reduce the postoperative morbidity as well as the surgical trauma to the patient. Among these grafting materials allogeneic bone substitutes are widely used worldwide. Numerous in-vitro and in-vivo investigations regarding the safety and predictability of those materials are available [12–16].

Allogeneic materials are tissues taken from individuals of the same species providing the advantage to be readily available without a second surgical site and are commercially available as demineralized freeze-dried bone allograft (DFDBA), freeze-dried bone allografts (FDBA), demineralized allograft putties and in various configurations from cortical and cancellous bone as well as in preparations from single or multiple donors. Furthermore, bone allografts provide osteo-inductive as well as osteoconductive properties and become resorbed within a reasonable amount of time and promote new bone formation [16–18]. In Germany, allogeneic bone grafting materials are classified as pharmaceuticals and undergo highest quality control standards through validated harvesting, cleaning, and manufacturing processes. During intraoral bone augmentation procedures immunological reactions can be promoted and most likely triggered by the bone grafting material used. In organ transplantation as opposed to the insertion of allogeneic bone substitute material in implantology multiple preoperative tests prior to organ transplantation are necessary in order to match the donor and the organ recipient in order to prevent graft rejection [19].

However, in the case of allogeneic bone grafting materials it is highly unlikely to observe immunological reactions like graft rejection because those materials are treated in a multi-step chemical cleaning process in order to inactivate potential pathogens as well as immunologically potent molecules, which was reported by several scientific groups [20–22].

Nevertheless, it has been reported in the literature that immunologic reactions and even alloimmunization processes after allogeneic bone grafting procedures were observed [23–27]. The incidence and prevalence of such immunologic reactions to bone grafting materials is not exactly known, however it could be expected that it could be comparable to the incidence and prevalence of adverse reactions to drugs (ADR) or other materials which is assumed to be about 7 % [28–30]. Those reactions are classified into type A (predictable on-target reaction) and type B (unpredictable, off-target reactions) reactions, which is thought to account for about 15 % of ADRs [29, 31]. Those off-target reactions are mostly immunologically caused hypersensitivity reactions according to the type IV (Coomb and Gell classification) immune reaction towards a peptide or similar antigens and are dose dependent [32].

In order to test for such an allergic reaction several testing options are available and described in the literature. Among those tests, clinical phenotype testing, in-vivo, and in-vitro cell provocation tests are available, where either blood and tissue samples are investigated, or the antigen is directly applied to the person’s skin (prick or patch test) [33]. It is widely known that T-cells play a dominant role in cellular type IV hypersensitivity reactions, therefore the Lymphocyte Transformation Test (LTT) is a very elegant and scientifically profound method to test for type IV hypersensitivity reactions [34, 35]. The advantage is that in-vitro analysis is carried out with drawn blood without any provocation of the patient. This avoids the risk of iatrogenic sensitization of the patient. In this test, the activation and proliferation of the memory T cells, responsible for an immune response, is measured after in-vitro antigen-specific stimulation of isolated mononuclear cells. The test has been well studied for the detection of drug sensitization. The sensitivity and the specificity for this application for the detection of antigen-specific T cells as base for hypersensitivity reactions were confirmed by various scientific groups and was determined to vary between 58 and 89% for the sensitivity and between 93 and 100% for specificity, respectively [36–40].

The aim of the present study was to test the null-hypothesis that there are no statistically significant differences between the plasma LTT levels (primary outcome) at different time points (T1 – T3) of patients receiving an allogeneic bone grafting material dehydrated by freeze-drying and pooled from multiple donors (Maxgraft®, test group 1), or a solvent-dehydrated allogeneic bone grafting material that is harvested from a single donor (Puros®, test group 2). As a secondary outcome the null-hypothesis was tested that there are no statistically significant differences on the immunohistochemical detection of pro-inflammatory cytokines IL-1α, IL1ß and TNF-α and T-cell markers CD4, CD8 in biopsies of the test groups.

## Materials and methods

### Study design, participants, and collection of plasma samples

The publication was performed and written according to the CONSORT Guidelines for randomized controlled clinical trials [41]. Blood samples were collected from a total of 60 patients, 25 patients (test group 1) who received allogeneic bone grafting material (Maxgraft® Allograft Cancellous Granules (< 2 mm) (Botiss Company, Berlin, Germany, part of Straumann Group, Basel, Switzerland)), and 25 patients (test group 2) who received allogeneic bone grafting material (Puros® Cancellous particulate Allograft (0.25-1 mm) (Zimmer Biomet, Warsaw, IN, USA)) as well as from 10 patients who were treated with autologous bone grafts (control group) for intraoral guided bone augmentation procedures for lateral alveolar ridge augmentation (Seibert class I) prior to dental implant placement. The patients were recruited and treated in a single periodontal office in Hamburg, Germany, between September 2018 to February 2019. All patients were systemically and orally healthy non-smokers and had according to the medical history no prior contact with an allogeneic material. General exclusion criteria for enrollment in this study were skeletally immature patients, persons with uncontrolled systemic diseases, a history of radio- and/or chemotherapy, immune suppression, pregnancy, active periodontal disease, poor oral hygiene, and smoking habit. Site-specific exclusion criteria were alveolar ridges where defects in bone height (Seibert class II and III defects) were present. At the time of enrollment in this study the patients were randomly assigned to five blocks of twelve participants each. A blinded clinician not involved in this study and not involved in the periodontal office, allocated the participants according to a computerized random number generator to one of the parallel groups by drawing numbered, opaque, and sealed randomization envelopes indicating the enrollment into test groups 1 and 2 (five participants each block) or the control group 3 (2 participants per block). The autogenous control group was intentionally reduced to 10 participants in order to reduce resources and costs of the study. The main intention of the autologous control group was to verify the precision and the accuracy of the LTT and to demonstrate that a positive test result could not be provoked e.g. by the surgical procedure itself.

**Fig. 1:**
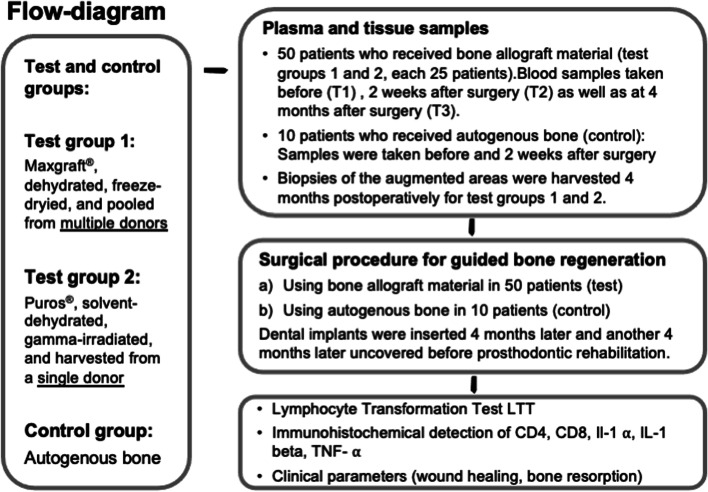
Flow diagram of study design. The flow-diagram shows the study design, the bone allograft material as well as the three different time points for blood sample collection and tissue biopsy as well as the subsequent laboratory experiments. Furthermore, the clinical procedure for the test and the control groups are shown

Blood samples for the test groups were collected on the day of surgery preoperatively (T1), 2 weeks postoperatively (T2), and 4 months postoperatively (T3). Blood samples for the control group were taken preoperatively (T1) and 2 weeks postoperatively (T2) for the above mentioned reasons (Fig. 1). All samples and data were anonymized by a blinded clinician not involved in this study and not involved in the periodontal office. The mean age of allograft patients of group 1 was 58 years (range: 39–78), and 56 years (range 28–73) for test group 2, and for autograft patients (control) it was 55 years (range: 32–76) (Table 1). The collection of the blood samples and experiments were performed in compliance with the World Medical Association Declaration of Helsinki (version 2008) and were approved by an ethics committee (Hamburg Medical Association, Germany, no. PV5211) and the study was registered with the German Register for Clinical Trials (DRKS00013010 on 30/07/2018). All patients gave their informed consent for participation in this study and for publication of the results, images, and pseudonymized data. All patients completed the study successfully and were available for follow-up and reevaluation visits once a year in the periodontal office where the surgical treatment was performed as well as in the general dental office where the prosthodontic rehabilitation was incorporated. No adverse events were recorded for the test groups and the control group 12 months postoperatively, however, the follow-up visits will continue in the future.

**Table 1:** Patient demographics and characteristics

	Maxgraft® (***N*** = 25)	Puros® (***N*** = 25)	Control (***N*** = 10)
**sex, n**			
**male**	15	12	7
**female**	10	13	3
**age, years**			
**mean ± SD**	58.1 ± 11.4	56.3 ± 13.4	55.2 ± 9.5
**min; max**	38.8; 78.3	28.8; 73.6	31.8; 76.3
**treated region, n**			
**maxilla**	17	18	5
**mandible**	8	7	5
**treated tooth/teeth, n**			
**anterior**	6	8	2
**posterior**	19	17	8
**healing period, months**			
**mean ± SD**	4.3 ± 1.1	4.2 ± 0.9	4,1 ± 0.7
**min; max**	4.0; 5.0	4.0; 4.8	4.0; 4.7

*SD* Standard deviation, *min* Minimum, max Maximum

Table 1 shows the patient demographics and characteristics regarding sex, age, treated region in the mouth and healing period in months

### Surgical procedure

Intraoral bone grafting for lateral alveolar ridge augmentation of Seibert class I defects was performed under local anesthesia using Ultracain-DS Forte (Sanofi-Aventis, Frankfurt/Main, Germany). After deflection of a mucoperiosteal flap a cortical perforation was carried out and particulate allogeneic bone grafting material (test groups 1, 2) or autogenous bone (control group) was inserted. The bone grafts were covered with a collagen membrane for guided bone regeneration, according to the manufacturer’s recommendations (Jason® membrane, Botiss Company, Berlin, Germany, part of Straumann Group, Basel, Switzerland). Prior to surgical closure, the bucco-lingual width of the grafted alveolar ridge was measured using a sterilized precision dental caliper (Dental caliper 800/5, Otto Leibinger GmbH, Griesweg 27, 78,570 Mühlheim, Germany). A periosteal releasing incision of the mucoperiosteal flap was performed in order to mobilize the flap for a tension-free primary closure of the surgical site. Flap-fixation was performed using a horizontal and vertical mattress suture with 5.0 Goretex filaments (W. L. Gore & Associates GmbH, Putzbrunn, Germany). The postoperative regime required a 2.0% chlorhexidine rinsing solution for post-operative oral hygiene. Post-operative appointments were scheduled after 1–2 days, 2, 6, and 12 weeks following surgery. Sutures were removed 2 weeks after the bone augmentation procedure. Dental implants were inserted in an open flap approach after approximately 4 months of healing. The bucco-lingual width of the healed alveolar ridges was measured again using the same sterilized precision dental caliper before biopsies were taken from the previously augmented sites using a trephine bur with a core diameter of 3.0 mm (Komet Dental, Gebr. Brasseler GmbH & Co. KG, Lemgo, Germany) and a speed of 600 rpm with external cooling using sterile saline. Drilling was performed to a maximum depth of 6.0 mm exactly at the position where implant placement was planned using a surgical guide. Implants with a diameter of at least 3.3 mm and a minimum length of 8.0 mm (Straumann Group; Camlog GmbH, Wimsheim, Germany; Astra Implant System, Dentsply Sirona Implants, Mannheim, Germany) were inserted according to the manufacturer’s recommendations with a mean insertion torque of 35 Ncm*.* After implant placement, the mucoperiosteal flap was readapted and fixed with Goretex sutures as described above. Uncovering and prosthetic restauration of the implants was carried out after the healing period of approximately 4 months. Two-dimensional radiographs, using the paralleling technique with a Rinn holder (Dentsply-Rinn, 1301 Smile Way, York, PA 17404, USA) were taken immediately following the bone augmentation procedure, immediately after implant insertion, and after final prosthetic reconstruction in order to visualize the final result as a baseline for future radiographic comparison. All patients were followed-up for 12 months after completion of the final prosthetic restoration. All treatments were provided by a single surgeon (ÖS) within a specialty practice limited to periodontology and implant dentistry in Hamburg, Germany. In this specialty practice, the patients were treated in accordance with established guidelines. Clinical documentation of the surgical procedures was published in previous publications [42, 43].

### Clinical parameters

Clinical parameters around the implant restoration were obtained following implant restoration using a calibrated implant probe (Hu-Friedy Mfg. Co., LLC. European Headquarters Astropark Lyoner Str. 9. D-60528 Frankfurt am Main Germany, Global Headquarters, Chicago, Illinois, USA) for the recording of 6-point pocket probing depth (PPD) as well as bleeding on probing (BOP), and periimplant recession defects (REC), PI (plaque index), or BI (bleeding index). Clinical measurements were obtained by one experienced calibrated blinded clinician.

### Lymphocyte transformation test (Syn. Lymphocyte proliferation test)

10 ml of heparinized venous patient blood were processed by ficoll density gradient centrifugation to obtain peripheral blood mononuclear cells (PBMCs). After washing the cells with PBS (PAA Laboratories, Linz, Austria), the cell pellet was resuspended to obtain a cell count of 1 × 10^6^/ml in cell culture medium (RPMI 1640; PAA) supplemented with 2 mM L-glutamine (PAA), 100 μg/ml gentamicin (PAA) and 5% autologous serum. Subsequently, 2 × 10^5^ PBMCs were incubated with an aqueous digestion of the two native allogeneic bone grafting materials in 3 different doses, respectively, in a 96-well plate (Nunclon, Wiesbaden, Germany) for 6 days at 37 °C and 5% CO_2_ atmosphere. All stimulations were performed in triplicates. The cells were labeled with 3H-thymidine (1 μCi/ml, Hartmann Analytics, Braunschweig, Germany) 12 hours prior to cell harvest. A cell harvester (Wallac) was used to harvest cells on glass fiber filters (Wallac, Lund, Sweden). The incorporated 3H-thymidine activity was measured as “counts per minute” (cpm) using a solid phase beta counter (Wallac). For analysis mean values of the triplicates were calculated. The results for each stimulation dose were finally given as a stimulation index (SI; ratio of cpm of the culture with and without test material).

### Histology

Histological specimens were evaluated on the basis of established methods in bone histology and pathology [44] or own published methods [44]. Each biopsy sample was fixed by immersion in 4% buffered formaldehyde (Sörensen buffer) at room temperature (RT) for at least 1 d and subsequently decalcified for about 2 to 3 weeks in 4.1% disodium ethylenediaminetetraacetic acid (EDTA) solution, which was changed every 24 h. After hydration, tissues were dehydrated in an ascending series of ethanol and embedded in paraffin. Serial longitudinal sections of 2–3 μm were cut and representative slides were stained with hematoxylin-eosin (HE).

### Immunohistochemistry

Histological specimens were immunohistochemically evaluated on the basis of established methods in bone histology and pathology [44] or own published methods [44] as well as methods from the literature on certain parameters investigated in similar studies on the healing of bone replacement materials [45–47]. Representative slides from the median parts of the sample series were deparaffinized, rehydrated and rinsed for 10 min in Tris-buffered saline (TBS). Endogenous peroxidase was blocked in a methanol/H_2_O_2_ (Merck, Darmstadt, Germany) solution for 45 min in the dark. Sections were pretreated with PBS containing 1% bovine serum albumin (BSA) for 20 min at RT, digested with 0.4% pepsin for 10 min at 37 °C and then incubated with the primary antibodies in a humid chamber. The following markers were investigated: immunological markers CD4 and CD8 as T-cell markers and IL-1α, IL1ß and TNF-α as pro-inflammatory cytokines. Antibody binding was detected with the peroxidase-conjugated EnVision® anti-mouse system or the EnVision® anti-rabbit/anti-goat HRP-conjugated secondary antibodies (Dako, Glostrup, Denmark), diluted 1:50 and incubated for 30 min at RT. Peroxidase activity was visualized using diaminobenzidine (DAB) yielding a brown staining product and slides were counterstained with Mayer’s hematoxylin.

Specificity controls were run by (i) omitting primary antibodies and applying TBS or normal horse serum instead, (ii) omitting primary antibodies or bridge and secondary antibodies, respectively. Antibody details and incubation protocols are listed in Table 2.

**Table 2:** Antibody details and incubation protocols

Antibody	Isotype	Manufacturer	Incubation protocol
CD4	rabbit monoclonal	Abcam (Cambridge, UK)	HP, EDTA buffer, 1:50, o/n, RT
CD8	mouse monoclonal	Dako (Glostrup, Denmark)	HP, citrate buffer, 1:50, 1 h, RT
IL-1α	goat polyclonal	Santa Cruz (Dallas, USA)	1:50, o/n, 4 °C
IL-1ß	rabbit polyclonal	Abcam (Cambridge, UK)	1:100, o/n, 4 °C
TNF-α	mouse monoclonal	Santa Cruz (Dallas, USA)	1:100, o/n, 4 °C

*HP* Heat pretreatment, *o/n* overnight, *RT* Room temperature

Table 2 provides an overview of the antibodies used for immunohistochemical staining regarding isotype, manufacturer, and incubation protocols

### Histological and immunohistochemical evaluation

Histological specimens were evaluated qualitatively and semi-quantitatively on the basis of established scoring methods in bone histology and pathology [44] or own published methods [44] as well as methods from the literature on certain parameters investigated in similar studies on the healing of bone replacement materials [45–47]. The assessment was always performed in a blinded way by two independent, histologically experienced examiners on three different sections of the section series (central, lateral). The sections were analyzed using a light microscope (Leica Microsystems GmbH, Wetzlar, Germany). Three representative regions of interest (ROI) were determined at a lens magnification of 40x. These were always in the center of the area with proven bone substitute material and at two apical or coronal or lateral margins to the autochthonous tissue.

Infiltrates were semi-quantitatively evaluated according to the following scheme: 0 = none; 1 = loose infiltrates, disseminated or focal; 2 = dense, moderately extensive round cell infiltrates; 3 = extensive, dense round cell infiltrates with highly endothelial venules, edema, focal giant cells; 4 = pronounced inflammatory reaction including giant cells, necrosis.

Histochemical and immunohistochemical findings with purely cellular localization (CD4, CD8) were semi-quantitatively evaluated as follows: 0 = negative; 1 = weak; 2 = moderate; 3 = strong; 4 = very strong. Pro-inflammatory cytokines IL-1α, IL1ß and TNF-α, which were detectable both cellularly and extracellularly (connective tissue, bone matrix), were semi-quantitatively evaluated according to the following scheme: 0 = negative; 1 = detection only in cells (e.g., osteoblasts, fibroblasts); 2 = detection in cells as well as extracellular with onset of bone formation (osteoid); 3 = detection both in cells and in bone matrix/connective tissue; 4 = strong detection in cells and extracellular.

### Statistical analysis

Statistical analyses were performed using the SPSS software package version 24.0 (SPSS Inc. Chicago, IL, USA) and Stat Plus version 8 (AnalystSoft Inc., Walnut CA). A sample size of 11 patients per group (number of groups = 2, total sample size = 22 patients) was the required samples to be statistically significant with 80% power and at a significance level of 95% (accepted α error = 0.05). The mean difference between the groups Maxgraft® and Puros® was 0.187, the mean standard deviation was 0.256 and the mean effect size was 0.665. The statistical differences of the measured data in the groups Maxgraft® (25 patients), Puros® (25 patients) and 10 controls were calculated using ANOVA with Tukey’s HSD test for all pairwise comparisons that correct for experiment error rate. Two-sample comparisons were performed using Student’s t-test for equal or unequal variance and Pearson linear correlation test where appropriate. Due to the small size of the variables, we categorized the immunohistochemical results into category 0 (stage: 0, 1) and category 1 (stage > 1), respectively. The Holm-Bonferroni method for multiple test correction was not applied. Missing data were handled by pairwise deletion. A *p*-value ≤0.05 was considered as statistically significant. All *p*-values are two-sided.

For the evaluation of the LTT results we performed a numerical evaluation. All < 2 data are considered to equal 1 (half of the average of the lower detection limit i.e. The average of the maximum (2) and the minimum (0)). We performed the Kruskal-Wallis test (non-parametric ANOVA) followed by Dunn’s multiple comparison test and two-way-ANOVA followed by Bonferroni’s test. *P* < 0.05 is considered a significant difference.

Furthermore, we performed an all or none evaluation. All < 2 data are considered negative and all > 2 are considered positive. Chi square test *P* < 0.05 is considered a significant difference (Supplementary material).

## Results

### Patient demographics and characteristics

Patient demographics and characteristics are summarized in Table 1. The mean age of allograft patients of group 1 was 58 years (range: 39–78), and 56 years (range 28–73) for test group 2, and for autograft patients (control), it was 55 years (range: 32–76), respectively. Test group 1 consisted of 15 male and 10 female patients while test group 2 included 12 male and 13 female patients, and the control group consisted of 7 male and 3 female patients. In the majority of patients, the maxilla was treated, and treatment of posterior areas was more common than treatment of anterior areas. There were no statistically significant differences in the regions where the biopsies were harvested, the age or the gender of the patients between or within the test and control groups. All 60 patients completed their treatment and participated at the follow-up treatment. No adverse events were observed.

### T- cell sensitization measured by LTT

**Fig. 2:**
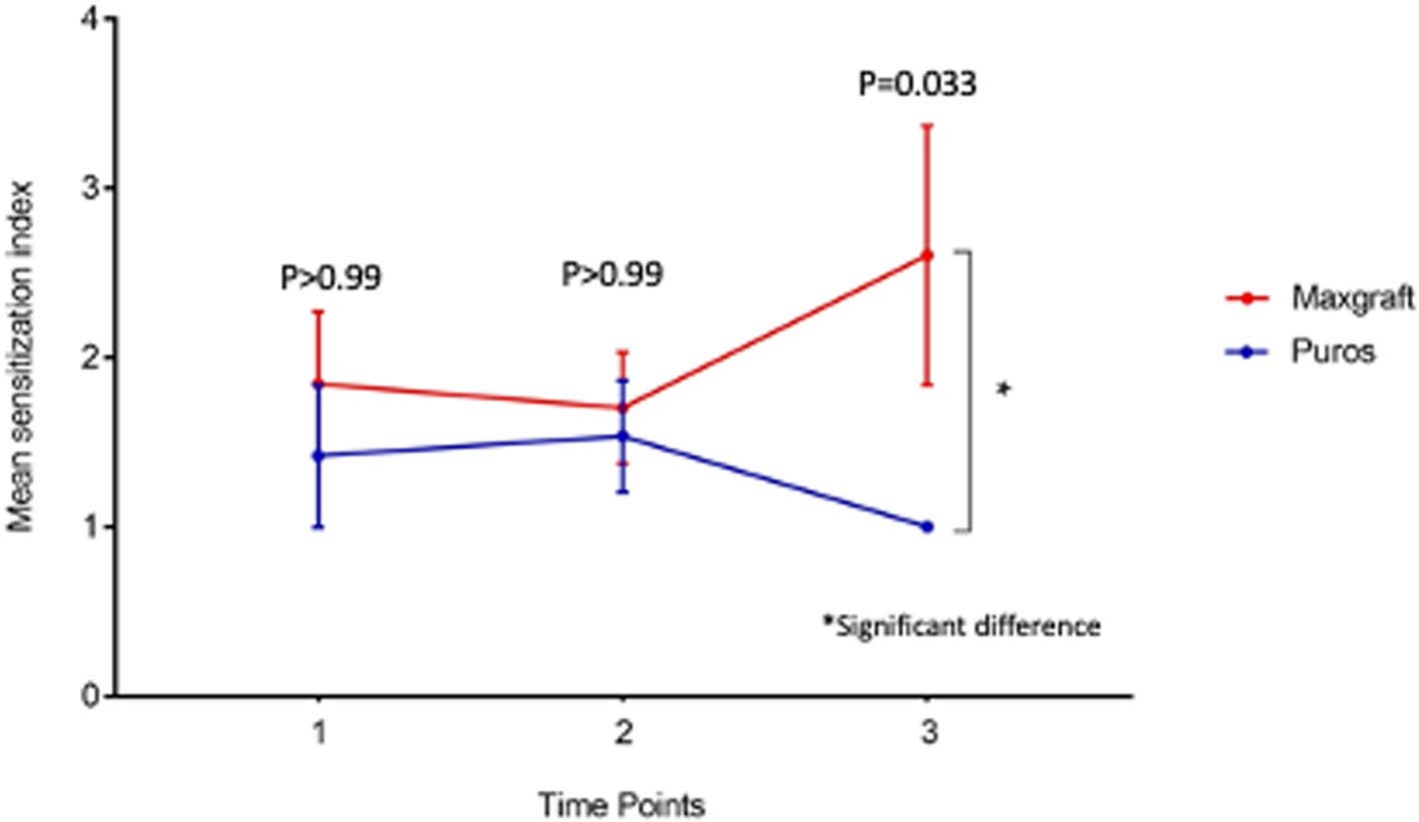
The fig. 2c shows the mean value of the stimulation indices for the 25 patients in the test group 1 (Maxgraft**®**, red) and the 25 patients in the test group 2 (Puros**®**, blue) for all 3 time points.  For calculation and presentation, all patients with a stimulation index in the LTT < 2 were set to 1. At time point 3, T-cell sensitisation to the material used is significantly more frequent in the Maxgraft**®** group (*p* = 0.033)

The detection of specific T-cells against components of the bone substitute materials was carried out in 60 patients. The result was given as stimulation index in order to account for the interindividual variations of the background proliferation. An SI value of > 2 is considered positive. We statistically analyzed differences between both tested materials for the LTT at the different timepoints T1, T2, and T3, respectively. At T1, we detected four positive results for test group 1 (Maxgraft®) and only one positive result for test group 2 (Puros®). At T2, we detected five positive results for test group 1 and three positive result for test group 2. At T3, we detected seven positive results for test group 1 and only one positive result for test group 2 (*p* = 0.033) Fig. 2. The differences for timepoints 1 and 2 were not significant (Fig. 2).

Furthermore, we detected three different patient groups of T-cell sensitization, group A demonstrated a negative LTT at T1-T3. Group B demonstrated a positive LTT at T1-T3, and group C demonstrated a negative LTT at T1 which became positive at T2 (sensitization occurred, Table 3).

**Table 3:**
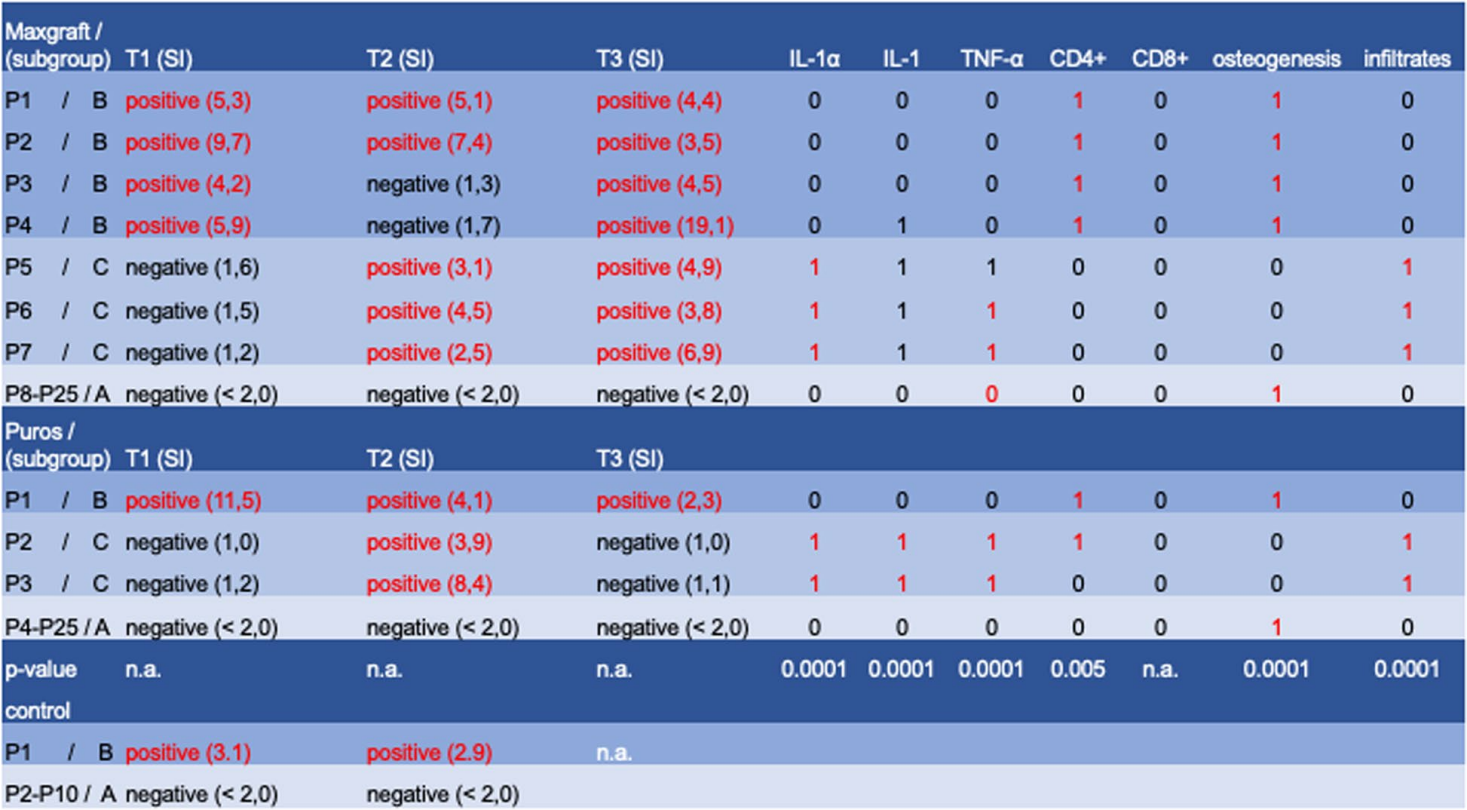
Immunological, histological, and immunohistochemical findings with statistical correlations

Tables 3 provides an overview of the immunological, histological, and immunohistochemical findings for the two test groups (Maxgraft and Puros) and for the immunological findings of the control group (autologous) and shows the statistical correlations of these parameters for the test groups and the corresponding subgroups A, B, and C.

For test group 1 (Maxgraft®) 25 patients were evaluated. Among those, 4 patients (16%) already demonstrated a positive LTT result preoperatively (group B) (SI range: 4.2–9.7; mean: 6.28, Table 3). Two of those patients lost this positive result interim following contact with the material at T2, which became positive again at T3 (SI range: 3.5–19.1; mean: 7.7, Table 3). Three patients (12%) which were LTT negative pre-operatively developed a positive LTT result after first contact with the material (group C) which remained positive throughout the observation period (SI range: 3.8–6.9; mean: 5.2 Table 3). The remaining 18 patients (72%) did not demonstrate a positive LTT result at any timepoint (group A, Table 3).

For test group 2 (Puros®) the same number of 25 patients were evaluated. Among those, 1 patient (4%) demonstrated a positive LTT result preoperatively (group B; SI: 11.5, Table 3). Two patients (8%) developed a positive LTT result after first contact with the material (group C; SI range: 3.9–8.4; mean: 6.1), which disappeared throughout the observation period at T3. The remaining 22 patients (88%) did not demonstrate a positive LTT result at any timepoint (group A, Table 3).

For the control group 10 patients were evaluated. Among those, 1 patient demonstrated a positive LTT result preoperatively at T1, which remained positive at T2 (group B; SI range: 2.9–3.1; mean: 3.0, Table 3). The remaining 9 patients (90%) did not demonstrate a positive LTT result at any timepoint (group A, Table 3). Those findings of the control group confirm the accuracy and the precision of the LTT and demonstrates that a positive LTT is not provoked e.g. by the surgical procedure itself.

Due to the small population size and in order to avoid false-significant *p*-values, we did not carry out a statistical comparison between the small sub-cohorts of group B and group C for the immunological findings. In order to verify whether these values would be statistically significant, further investigations with larger population sizes are planned in the future.

### Histological and immunohistochemical findings

Shortly, all biopsies showed osteogenesis in different stages by forming mostly cancellous woven bone around allogeneic remnants. Remodeling into mature lamellar bone was observed in some specimens. Intertrabecular regions consisted of loose or fibrous tissues. Osteoclastic resorption could not be found.

Due to the small size of the variables, we categorized the immunohistochemical results into category 0 (stage: 0, 1) and category 1 (stage > 1), respectively in order to carry out a statistical comparison between the small sub-cohorts groups A, B, and group C.

The occurrence and semi-quantitative evaluation of infiltrations and immunohistochemical findings for CD4 and CD8 have already been investigated [43]. However, those findings were not correlated to the immunological findings of the LTT results, which is substance to the present study. CD4-positive lymphocytes were seen in five of the specimens investigated, all belonged to LTT group B, CD8-positive cells within an infiltration in only one case (Table 3). In five specimens of group B from both materials areas of dense infiltrations consisting of round cells were located in the intertrabecular connective tissue or at the periphery. The statistical analysis of the categorized semi-quantitative evaluation of the detection of CD4 positive cells in group B of both materials revealed a highly significant correlation (*p* = 0.0005). Furthermore, the statistical analysis of the categorized semi-quantitative evaluation of the occurance of infiltrates in group C of both materials revealed a statistically highly significant correlation (*p* = 0.0005). In those specimens, a very low score for osteogenesis of 0 or 1 was observed (Table 3), however, a statistically significant result was only observed for group C (*p* = 0.005).

**Fig. 3:**
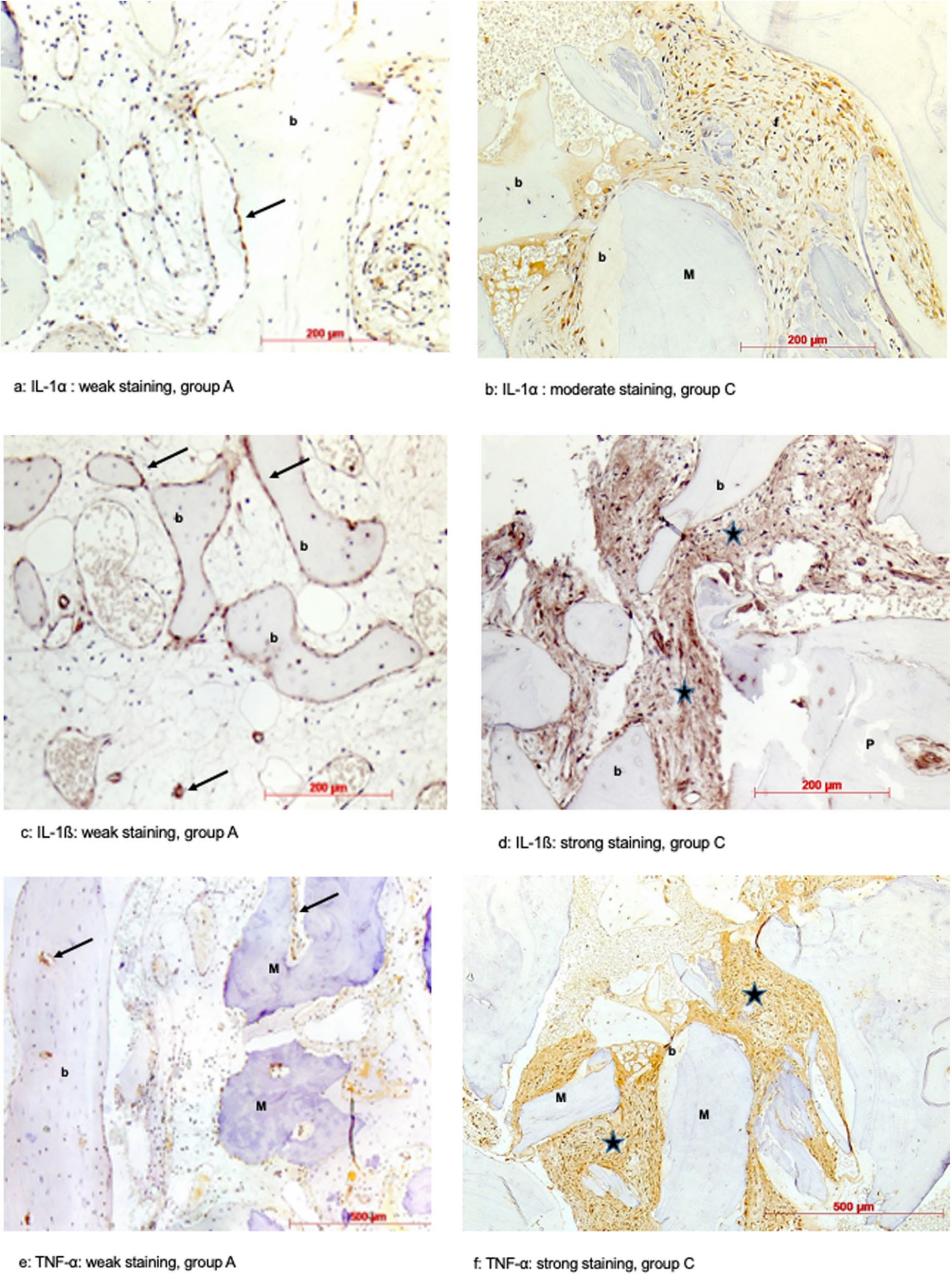
**a-f** Immunohistochemical analysis of IL-1α, IL-1ß and TNF-α. a, b Immunostaining for IL-1α. (A) Weak immunostaining pattern: staining of bone lining cells (arrow), b = newly formed bone, Maxgraft**®**, patient of group A, DAB, original magnification × 20); (B) Moderate immunostaining: Staining of a cluster of fibroblasts (f), b = newly formed bone, M = Maxgraft**®** granules, Maxgraft,**®** patient of group C, DAB, original magnification × 20. **c**, **d** Immunostaining for IL-1ß. (A) Weak immunostaining pattern: staining of bone lining cells and vessel walls (arrows), b = newly formed bone, Puros**®**, patient of group A; DAB, original magnification × 20); (B) Strong immunostaining: stronger extracellular immunoreactivity within connective tissue areas (asterisks), b = newly formed bone, Puros**®**, patient of group C, DAB, original magnification × 20. **e**, **f **Immunostaining for TNF-α. (A) Weak immunostaining pattern: staining of fibroblasts and vessel walls (arrows), b = newly formed bone, M = Maxgraft**®** granules, patient of group A DAB, original magnification × 10); (B) Strong immunostaining: Extracellular immunoreactivity and fibroblast staining (asterisks) in the connective tissue among Maxgraft**®** granules (M) with newly formed bone (b), patient of group C DAB, original magnification × 10

IL-1α immunostaining was negative or weak in most cases. Weak staining appeared as focally immunoreactive fibroblasts, bone lining cells or lymphocytes. In one case of group C moderate staining and in four cases of group C a strong staining appeared, which was characterized by larger clusters of immunoreactive cells and moderate to strong extracellular reactivity mostly in areas with bone substitute remnants or infiltrations. The statistical analysis of the categorized semi-quantitative evaluation of the detection of IL-1α in group C of both materials revealed a highly significant correlation (*p* = 0.0001). There was no difference between allograft materials observed (Table 3; Fig. 3 A, B).

A similar immunoreactive pattern as for IL-1α could be observed for IL-1ß. However, there were more cases with strong staining. In these cases, stronger extracellular staining and immunoreactivity of more cells were visible. All cases of moderate to strong staining appeared in group C. The statistical analysis of the categorized semi-quantitative evaluation of the detection of IL-1ß in group C of both materials revealed a highly significant correlation (*p* = 0.0001). There was no difference between allograft materials observed (Table 3; Fig. 3 C, D).

TNF-α immunoreactivity was mostly negative or weak in Puros specimens except for the two cases of group C, whereas among the Maxgraft specimens one moderate and even two strong cases (group C) could be seen. Weak immunoreactivity was restricted to some fibroblasts, cells in infiltrations, bone lining cells and vessel walls. Larger areas of additional extracellular staining were characteristic for specimens with moderate and strong immunoreactivity. The statistical analysis of the categorized semi-quantitative evaluation of the detection of IL-1ß in group C of both materials revealed a highly significant correlation (*p* = 0.0001). There was no difference between allograft materials observed (Table 3; Fig. 3 E, F).

### Clinical findings

All patients completed the study and no severe adverse events were observed. All patients demonstrated a sufficient bone volume for implant placement following bone augmentation procedures with allogeneic bone grafting materials (test groups 1 and 2), and autologous bone (control group). However, a delayed wound healing was observed in 3 patients of test group 1 (Maxgraft®) and 2 patients of test group 2 (Puros®), all 5 patients belonged to LTT group C. Those patients also demonstrated a higher amount of resorption of the bone grafting material, which was confirmed at time of implant placement by bucco-lingual measurement and needed to be re-augmented at time of implant placement. At the time of implant placement and second bone augmentation, no delayed wound healing or any other complications were observed in these patients. Furthermore, no complications during wound healing could be observed in all other patients of test group 1 and 2 or the control group.

There were no differences within groups or between groups for any other clinical parameter like BOP (bleeding on probing), PPD (probing pocket depth), REC (gingival recession), PI (plaque index), or BI (bleeding index) within the follow-up period of 12 months following completion of the final restoration.

### Correlation between immunological, immunohistochemical, and clinical findings

As mentioned before, the planned implant restoration could be completed in every patient included in this study. However, the clinical observations of delayed wound healing and in some cases even the need for a second bone augmentation procedure for patients belonging to group C were supported by statistically significant correlations of the categorized parameters between the histological detection of infiltrates in the biopsies of patients belonging to group C of both materials (*p* = 0.0001) as well as a strong correlation of reduced osteogenesis in those patients (*p* = 0.0001) of group C for both materials. Furthermore, we observed a statistically significant correlation of the categorized parameters of the immunohistochemical detection of pro-inflammatory cytokines like IL-1α, IL-1β, and TNF-α for the patients of group C, who developed a positive LTT result at T2, after being negative at T1. Interestingly, the positive LTT result at T2 for patients of group C disappeared for the Puros® patients at T3 and remained positive for the Maxgraft® patients. Again, due to the small population size and in order to avoid false-significant *p*-values, we did not carry out a statistical comparison in between the small sub-cohorts of group B and group C for the immunological findings. However, following implant placement surgery no further delayed wound healing or any other adverse events were detected (Table 3).

For patients of group B (positive LTT from T1-T3) a statistically significant correlation of the categorized parameters for the immunohistological detection of CD4 positive cells in those biopsies (*p* = 0.005, Table 3) was observed. Clinically, there was no difference of wound healing or any other parameters observed compared to patients of group A (negative LTT).

## Discussion

In the present study, we determined a cellular sensitization to allogeneic peptides present in the bone substitute materials tested using the LTT and investigated the corresponding histological, immunohistochemical and clinical findings in patients receiving two different allogeneic bone grafting materials and a control group receiving autogenous bone grafts. To our knowledge, this investigation represents the first prospective randomized controlled clinical trial in humans evaluating these specific correlations, however, HLA-sensitization as well as the detection of donor cells and DNA in allogeneic block grafts was previously reported in the literature [27, 48–50]. In a previous RCT we compared the histologic and immunohistochemical findings of these two different allogeneic bone grafting materials (Maxgraft**®** and Puros**®**) regarding osteogenesis and graft resorption as well as the presence of major histocompatibility complexes (MHC) in the allografts itself [43]. Those findings did not show any significant differences between the two tested materials. Furthermore, we measured the soluble protein content in the tested graft materials revealing concentrations ranging from 0.38–1.50 μg/mg dry mass (Maxgraft®) and 0.47–1.70 μg/mg dry mass (Puros®), however, we did not detect any evidence for the presence of MHC I in those materials using ELISA analysis [43]. Other research groups, however, reported that MHC molecules have been detected in some but not all allogeneic bone blocks processed by peracetic-acid-ethanol-sterilization (PES) and postulated that the presence of such remnants may have a significant impact on graft incorporation and long-term survival of the graft [27]. Furthermore, other investigators observed a HLA-sensitization in 33% of the investigated patients following intraoral bone grafting with fresh-frozen allogeneic block grafts [48, 50] However, this group did not detect any associations with increased graft resorption after 6 months [49]. The safety and predictability of allogeneic bone grafting materials was investigated and confirmed in several publications [15, 51, 52]. Since the clinical survival rate for dental implants placed following intraoral bone augmentation procedures using bone allogeneic grafting materials varies between 82.8–90.9% for an observational period of 3–5 years, it remains uncertain which factors may influence this survival [53]. One possible factor could be an individual immunological reaction of an individual patient towards remnants of potential immunogenic molecules within the material. In the present study, we identified three different groups of patients regarding their LTT responses at different timepoints. The majority of patients did not show any positive response towards the grafting material (group A). One group of patients did demonstrate a positive LTT result towards the allograft materials even preoperatively (group B) and most interestingly, one group developed a positive LTT result following contact with the allogeneic material (group C). This group C consisted of very few patients in our study and counted only for 12% (3 of 25 patients for test group 1, Maxgraft**®**) and 8% (2 of 25 patients for test group 2, Puros**®**). As mentioned above, for patients of group B (positive LTT from T1-T3) a statistically significant correlation of the categorized parameters for the immunohistochemical detection of CD4 positive cells in those biopsies (*p* = 0.005, Table 3) was observed. These findings could be explained for group B in the context, that there is a significant HLA mismatch between the donor or donors and the recipient. This may be due to excessive genetic differences alone and may be exacerbated by exposure to this bone replacement materials previously. However, a previous contact to the materials tested was excluded prior to inclusion in this study. A sensitization to allogeneic peptides could also be induced by another way of contact with an allogeneic material, e.g. blood transfusion or organ transplant. However, this was not possible to confirm through medical history.

Most strikingly, the patients of group C seemed to develop a sensibilization towards the bone allograft through their first contact with the material. This sensibilization most likely represents a type IV immune reaction, which could be tested by the LTT. This type IV immune reaction is a T- cell mediated immune response and consists of activation of antigen-specific T-helper cells and the development of specific T- memory cells [54]. Mainly T-helper 1 cells are related to an immune response of type IV and they are able to activate macrophages causing secretion of co-stimulating pro-inflammatory cytokines, such as IL-1α, IL-1β as well as TNF-α [54].

In the present study, we detected a statistically significant correlation of the categorized parameters of the immunohistochemical detection of the pro-inflammatory cytokines IL-1α, IL-1β, and TNF-α for the patients of group C, who developed a positive LTT result at T2, after being negative at T1 (*p* = 0.0001). Furthermore, a statistically significant histological detection of infiltrates (*p* = 0.0001) as well as a reduced osteogenesis was observed in this group of patients (*p* = 0.0001, Table 3).

This finding could be explained that a sensitization might be triggered by an activation of T-helper 1 cells as well as an interaction with MHC II caused by the allogeneic material. Those findings were partly confirmed by an in-vitro study by Horowitz et al. [55] in which the induction and proliferation of T-cells was induced by MHC I and II determinants of the allogeneic bone. They proposed that pro-inflammatory cytokines, like IL-1α, IL-1β, and TNF-α are involved in this response. However, this group did not further investigate the cytokine response associated with their in-vitro findings. In contrast to our findings, they did not detect any CD4+ cells, only a strong CD8+ response was confirmed. They explained this result with the fact that CD8+ killer cells were able to remove the major source of the allo-antigen, the allogeneic cells, but this could also be due to different cut off values to delineate positive and negative results. They also postulated in their study that this could be associated with graft failure, modification of bone biology, including remodeling, wound healing and incorporation of the allograft, comparable to earlier findings of Bonfiglio et al. [56]. However, sensitization is not always accompanied by a T-cell mediated immune response, and even if this does occur, it does not always lead to clinically manifest inflammation. Therefore, it is not surprising that in various studies these allogeneic materials have proven to be very bio compatible [12–15].

As already discussed in Solakoglu et al. (2019) [43], an immune-inflammatory response may be a prerequisite for the integration of biomaterials including bone substitute materials into mature bone as already known for fracture healing or bone regeneration in general [57]. Inflammatory cell responses and upregulation of cytokines, e.g. TNF-α or IL-1, has also been investigated for osteoconduction of calcium phosphate bone substitutes [58, 59]. The immunochemical detection of cytokines, as demonstrated in the present study should be seen on the background of a modulation of the immune microenvironment during bone substitute healing, where macrophage subtypes and cytokines may promote osteogenetic effects [60].

To the best of our knowledge, no study regarding the investigation of allogeneic bone substitute using the LTT is available in the literature. Pinkowsky et al. investigated in their prospective clinical trial, the human lymphocyte reaction in freeze-dried allograft and xenograft ligamentous tissue using the lymphocyte blast transformation test and a corresponding SI of > 3 accounting for interindividual variances [60]. They found a positive SI in 75% of patients receiving a xenograft and in 50% of patients receiving an allograft. One of the allograft patients had such a severe reaction, that the graft had to be removed. None of the control patients receiving an autograft demonstrated a positive SI. Within the limits of that study, in which only eight individuals per test group and four patients in the control group were enrolled, the high amount of positive results of 75% for xenograft and 50% for allograft is very concerning. However, the clinical outcome was only harmful in one patient.

It is difficult to compare those results because of the different methods of graft preparation prior to clinical use. In the above mentioned studies freshly harvested as well as freeze-dried allogeneic tissue was used. During the freeze-drying process, a high amount of antigenicity is removed, however, there still remains a major concern regarding the transmission of viral diseases as well as graft rejection due to development of allo-antibodies, most likely caused by the transmission of donor cells and DNA [48, 61, 62]. These concerns are mostly excluded in the allogeneic materials used in the present study since both bone grafting materials are treated in a multi-step chemical cleaning process to inactivate potential pathogens. Maxgraft® is a pooled allogeneic material from multiple donors and finally dehydrated by freeze-drying, whereas Puros® is an allogeneic material harvested from one single donor per batch and is dehydrated using a solvent dehydration process prior to packaging and gamma-irradiation. Each process has been validated to inactivate viruses and bacteria and preserve the natural collagen-bone mineral composition which prevents disease transmission by removing and/or inactivating cells, viruses, antigens, and pathogens [14]. However, the complete elimination of viruses and bacteria does not protect against a low residual content of potential antigenic allogeneic material. The complete elimination of antigenic material can probably not be achieved in 100 % of cases and if a strong individual sensitization is present, the smallest amounts of antigenic materials might be sufficient to elicit an immune response.

Therefore, it is very noticeable that we were able to detect positive LTT responses in the patients in our present prospective randomized controlled clinical trial. However, we are aware of the fact that the relatively low sample size of 25 patients per test group is a limitation and does not allow for general conclusions on these materials. However, those correlations and associations need to be confirmed in future studies with larger patient populations.

Nevertheless, we were able to identify three different subgroups in both materials tested. The clinical correlations demonstrated no differences in wound healing between groups A and B. Group C of both materials demonstrated a comparable delayed wound healing and group C of test group 1 (Maxgraft®) demonstrated a higher amount of bone resorption which made a second bone grafting procedure at time of implant placement necessary. This difference in bone resorption was also observed in another recently published prospective randomized clinical trial [63]. Again, there was no statistically significant difference detectable between both materials, which was most likely due to the low sample size, but a strong clinical association was observed.

Furthermore, in group C of the test group 1, the positive LTT result remained throughout the observational period, whereas for test group 2, it disappeared after 4 months and the LTT became negative again at T3 (*p* = 0.033). This could probably be explained by the fact that in individual cases, permanent contact with the antigenic material leads to the development of immunological tolerance. One reason that might explain the small difference between both materials may be the fact that material 1 (Maxgraft**®**) is pooled from multiple donors and material 2 (Puros**®**) is harvested from a single donor. Postulating, that even after the very thorough and validated cleaning and preparation processes of both materials, a very low antigenic potential might remain, possibly leading to a slightly higher probability of immunological incompatibility by multiple donors than a single donor material.

In the present study for the detection of T-cell sensitization, the LTT was chosen in its classical form as an in vitro method. The epicutaneous test as a standard method could not be used in our study due to the lack of experience with the penetration of bone substitute materials through the skin as well as the lack of approved test preparations for the use in humans. There are various test modifications for the detection of in vitro induced T-cell activation available. These include cytokine-based and cytofluorometric methods in addition to the 3-H thymidine method used in this study. With these different methods, it is crucial that the test performing laboratory has sufficient experience with the method used. Therefore, in the present study it was decided to use the LTT as a 3-H-thymidine variant because this test has been accredited for many years in the test performing specialized laboratory according to DIN 15189, i.e. it is carried out according to a methodology standardized in house. This accreditation applies to the testing of drugs and of native materials, respectively. Even though cell culture methods with high sensitivity and specificity have been developed over the past 20 years, the limitations of those methods are the lack of automatization and the significant amount of time needed. Therefore, those methods are not practical to be used in a clinical study with a high number of participants.

However, false positive and false negative results cannot be excluded using the LTT method, the patch test, and other allergological sensitization tests and need to be taken into account as a limitation of those methods. Therefore, especially in cases of weak positive results the investigation of a parallel control group, as we carried out in the present study, is very important.

Furthermore, it should be emphasized that the detection of an immunological T-sensitization does not necessarily have to be accompanied by clinically relevant allergy symptoms in every case, because sensitization is only the prerequisite for an allergic reaction when the immune system comes into contact with the respective antigen.

## Conclusion

Even though immunological sensitization will not have a detectable clinical relevance in every case, according to the results of the present study it would be desirable to have a preoperative matching test available. This would allow to determine the compatibility of an allogeneic bone substitute material with the individual patient in order to increase patient safety and improve the predictability of these materials. We would suggest that in addition to the well-established tests that examine viral, bacterial and cellular remnants, immunological compatibility should also be considered. In the present study we used the LTT for the detection of immunological compatibility and our null-hypothesis for the primary outcome was rejected because there were detectable differences between the LTT levels of patients of the test groups at time point 3 (T3). Furthermore, the null-hypothesis for the secondary outcome was rejected because there were statistically significant differences detected on the immunohistochemical detection of pro-inflammatory cytokines IL-1α, IL1ß and TNF-α and T-cell markers CD4, CD8 in biopsies of the different subgroups identified for the test groups (group A, B, and C).

However, it would be desirable to include greater numbers of participants using a similar study design, even in a multi-center approach, in order to verify our results and to possibly develop a preoperative strategy to identify potential risk factors prior to allogeneic bone grafting.

## Supplementary Information

**Additional file 1.** Numerical and all-or-none statistical analysis of the tested materials.

## Data Availability

The authors declare that all relevant data supporting the findings of this study are included within this article. For further requests, the corresponding author (ÖS) should be contacted.
