# Supplementary material for: Characterization of immunologically detectable T-cell sensitization, Immunohistochemical detection of pro-inflammatory cytokines, and clinical parameters of patients after allogeneic intraoral bone grafting procedures: a prospective randomized controlled clinical trial in humans
Source: BMC Oral Health. 2022 Dec 10;22:592. doi: 10.1186/s12903-022-02584-6 (PMC9741780; doi:10.1186/s12903-022-02584-6)

Two statistical approaches:

A) Numerical: **All <2 data are considered to equal 1** (half of the average of the lower detection limit i.e. The average of the maximum (2) and the minimum (0)). **Kruskal-Wallis** test (non-parametric ANOVA) followed by Dunn's multiple comparison test and **two-way-ANOVA** followed by Bonferroni's test. P<0.05 is considered a significant difference.

B) All or none: All **<2** data are considered **negative** and all **>2** are considered **positive**. **Chi square test**. P<0.05 is considered a significant difference.

Numerical statistical analysis of the test materials (A, B) and comparison of the materials (C)

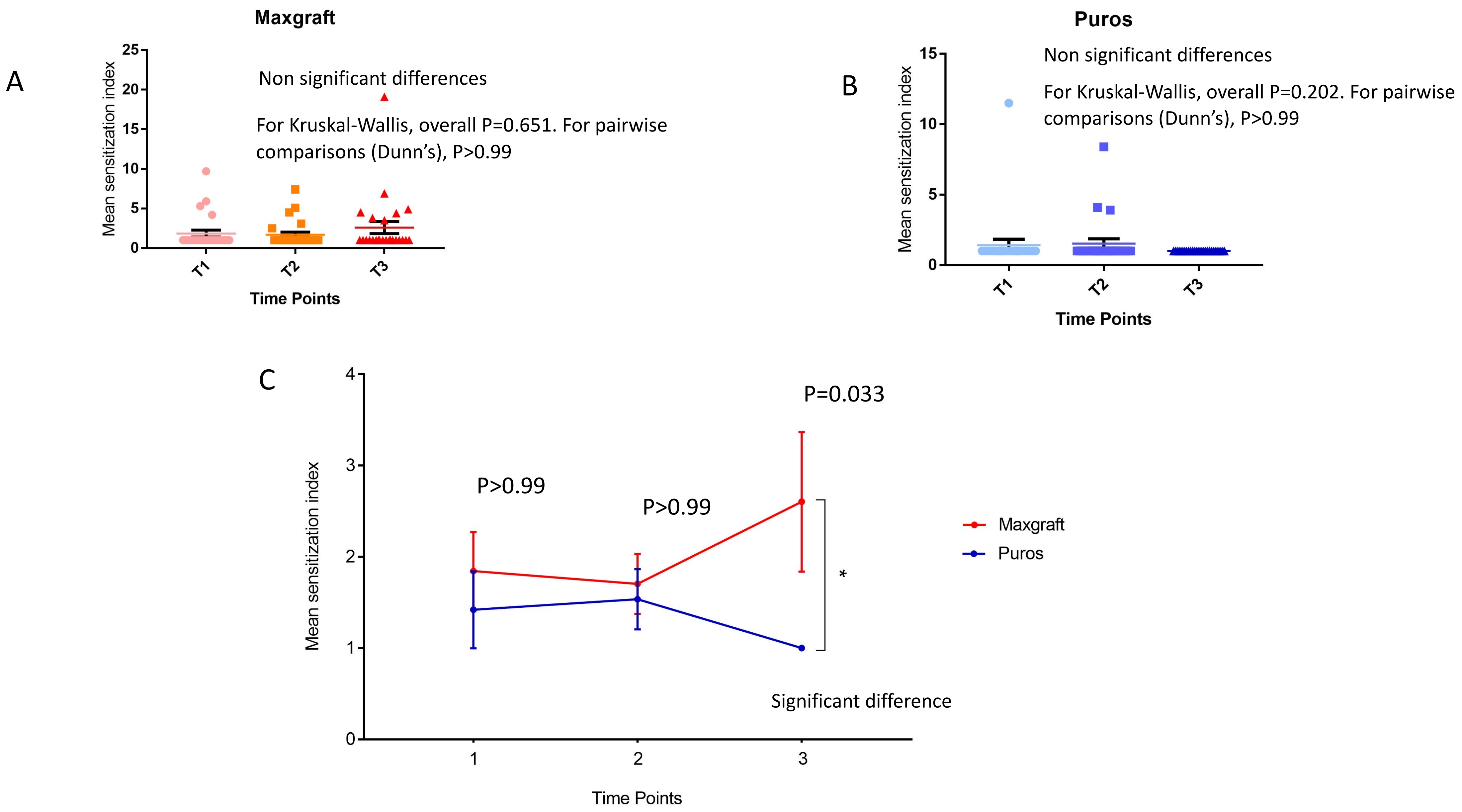

All-or-none statistical analysis of the test materials (D, E) and comparison of the materials F)

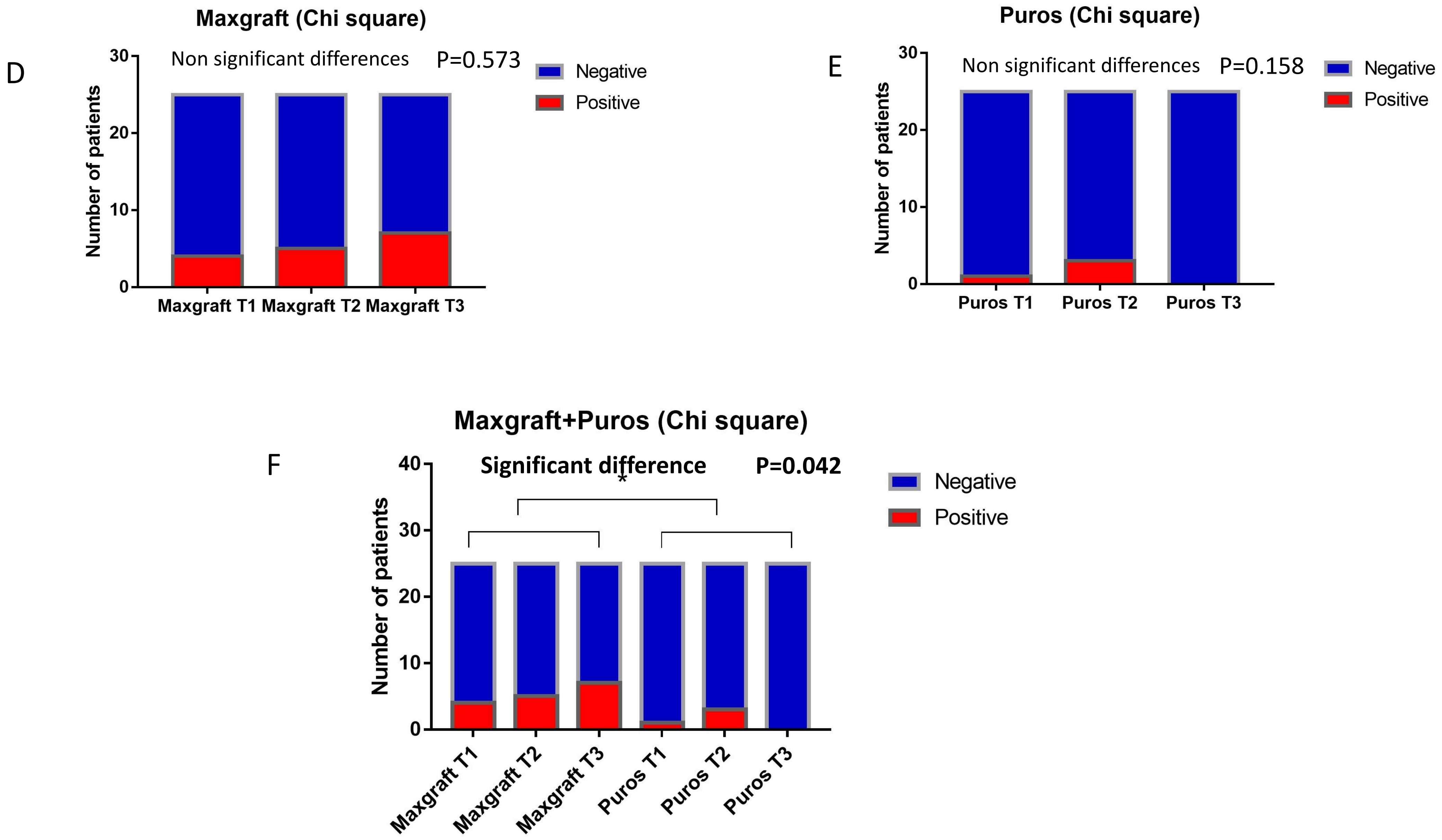

Supplement: Supplementary file 1 — Additional file 1. Numerical and all-or-none statistical analysis of the tested materials. [file 12903_2022_2584_MOESM1_ESM.pdf]
